# Combinatorial Evaluation of Corrosion Resistance of Ru-Based Alloys in Supercritical Acidic Fluids

**DOI:** 10.3390/ma19091844

**Published:** 2026-04-30

**Authors:** Rikito Murakami, Murugesan Naveenkarthik, Kei Kamada, Akira Yoshikawa

**Affiliations:** 1 Institute for Materials Research, Tohoku University, 2-1-1 Katahira, Aoba-ku, Sendai 980-8577, Miyagi, Japan; murugesan.naveenkarthik.t5@dc.tohoku.ac.jp (M.N.); akira.yoshikawa.d8@tohoku.ac.jp (A.Y.); 2 C&A Corporation, 1-16-23 Ichibancho, Aoba-ku, Sendai 980-0811, Miyagi, Japan; kei.kamada.c6@tohoku.ac.jp; 3 Graduate School of Engineering, Tohoku University, 6-6-40 Aramaki Aza-aoba, Aoba-ku, Sendai 980-8579, Miyagi, Japan; 4 New Industry Creation Hatchery Center, Tohoku University, 6-6-10 Aoba, Aramaki, Aoba-ku, Sendai 980-8579, Miyagi, Japan

**Keywords:** ruthenium alloys, corrosion-resistant alloys, high-throughput screening, combinatorial film deposition, supercritical acidic fluid

## Abstract

**Highlights:**

- An efficient combinatorial approach was established for alloy screening in supercritical acidic flow environments.
- Corrosion resistance of Ru–Mo–W and Ru–Mo–Fe–Cr alloys in supercritical nitric acid was systematically investigated.
- Highly corrosion-resistant compositions (<0.2 mm/y) were identified even at Ru contents below 60 at.%.

**Abstract:**

Ruthenium (Ru) and its alloys exhibit high corrosion resistance in acidic environments; however, their corrosion behavior in supercritical acidic fluids remains unclear. In this study, the corrosion resistance of Ru–Mo–W and Ru–Mo–Fe–Cr alloy systems in supercritical nitric acid was evaluated using a combinatorial approach. Alloy thin-film libraries with a thickness of approximately 300 nm were fabricated on (001) sapphire substrates by multi-target magnetron sputtering. Composition and thickness were evaluated by X-ray fluorescence (XRF) before and after corrosion testing. The composition-dependent corrosion behavior was compared in a 0.2 M nitric acid solution at 400 °C and 30 MPa. In the Ru–Mo–W system, most Ru-rich compositions exhibited corrosion rates below 0.2 mm/y, and at comparable Ru contents, Mo addition provided higher corrosion resistance than W addition. In the Ru–Mo–Fe–Cr system, significant dissolution and film delamination were observed on the low-Ru side. These results demonstrate that the combination of combinatorial thin-film libraries and XRF-based thickness evaluation is an effective first-stage approach for identifying promising alloy composition regions in supercritical acidic environments.

## 1. Introduction

Corrosion of structural materials has long been a major issue in technologies utilizing supercritical water (SCW) [1,2,3,4,5,6]. In supercritical water oxidation (SCWO), SCW is effective for the detoxification and volume reduction in wastes and sludge, and its application to the treatment of hazardous materials, including polychlorinated biphenyls (PCBs) and chemical warfare agents, has also been reported [1,2]. In addition, power generation using supercritical geothermal fluids, which is expected to serve as a next-generation power generation technology, is anticipated to provide much higher output per well than conventional geothermal systems. However, corrosion of casing pipes and surface facilities remains a serious challenge because the fluids are exposed to high temperature and high pressure and contain various ion species leached from underground formations, such as Cl^−^, SO_4_^2−^, HCO_3_^−^, and F^−^, and may also exhibit high acidity [3,4,5,6].

Under supercritical conditions (>374 °C and pressure > 22.1 MPa), the density, dielectric constant, ionic product, and salt solubility of water change markedly. As a result, the dissociation equilibria of acids, bases, and salts, the behavior of dissolved gases, and the stability of passive films also change, giving rise to corrosion mechanisms different from those under ambient conditions [7,8]. Consequently, corrosion modes such as general corrosion, pitting corrosion, and intergranular corrosion strongly depend on temperature, pressure, coexisting ion species, the presence or absence of oxidants, and flow conditions [7,8,9].

Stainless steels, Ni-based alloys (such as Inconel 625 and Hastelloy C-276), and Ti-based alloys have been widely studied as corrosion-resistant materials for SCW environments [10,11,12]. Recent studies have further examined the oxidation behavior of 316L stainless steel [13] and the superior oxidation resistance of high-Cr Ni-based alloys such as Alloy 690 in deaerated SCW [14], as well as material optimization for supercritical water oxidation of radioactive waste [15]. Despite these material-specific studies, SCW process environments exhibit wide variations in redox conditions, acidity, and ionic strength, making it difficult for a single material to maintain sufficient corrosion resistance under all conditions [11]. For example, under SCW conditions containing chlorides, phosphates, and oxidants, type 316 stainless-steel exhibits severe spallation of oxide scales [12]. Ni-based alloys are also promising candidate materials; however, their corrosion resistance strongly depends on alloy composition and the coexisting corrosive species, and their behavior changes markedly when Cl^−^, phosphates, and oxidants coexist [12,16,17]. Ti-based alloys are known to show relatively high corrosion resistance under similar conditions; however, when the Cl^−^ concentration is high, severe aqueous corrosion below the supercritical point has been reported [18]. Therefore, exploration of new corrosion-resistant alloys applicable to more severe supercritical oxidizing environments, such as supercritical geothermal systems, is required.

Ru is a relatively inexpensive platinum-group element and exhibits high chemical stability, although Ru alloys have generally suffered from poor room-temperature ductility [19,20]. In previous studies, the authors developed Ru–Mo–W-based alloys (Ruscaloy) with high strength and high toughness by adding Mo and W, and successfully fabricated long single-crystal wires (φ0.8 mm and >30 m in length) using the dewetting micro-pulling-down (μ-PD) method, which is a rapid single-crystal growth technique [21,22,23,24,25]. These Ruscaloy single-crystal wires were originally developed as high-efficiency, long-lifetime resistance-heating materials for vacuum evaporation in organic light-emitting diode fabrication. However, because they also exhibited high corrosion resistance, the corrosion behavior of Ruscaloy and related Ru–Mo–W–Fe alloys has been investigated, and excellent corrosion resistance against sulfuric acid and hydrochloric acid at 300 °C, as well as heated hydrofluoric acid, has been demonstrated [26]. Nevertheless, it has remained unclear whether the corrosion resistance observed under atmospheric pressure can be maintained under supercritical conditions.

Conventional supercritical corrosion tests have mainly relied on exposure tests using plate- or tube-shaped specimens. Because only a limited number of compositions can be evaluated in a single test, optimization of multicomponent alloy compositions requires a large number of trials. In contrast, combinatorial approaches based on composition-spread thin films formed on substrates are effective for accelerating materials exploration [27]. In corrosion research, such approaches have been applied to the microelectrochemical evaluation of Fe–Cr–Mo thin films [28], high-throughput electrochemical screening of Ni–Cr thin films [29], and optical-permeability-based screening of fast-degrading Mg–Zn libraries for biodegradable implant applications [30]. More recently, this strategy has been extended to corrosion-resistant alloy screening in the Ir–Ni–Ta system [31] and to high-throughput-to-high-fidelity screening of Al–Co–Cr–Fe–Ni compositionally complex alloys [32]. It has also been shown that thin-film libraries are effective for identifying relative compositional trends, although quantitative passivation behavior differs from that of bulk alloys [33]. In this study, therefore, Ru–Mo–W and Ru–Mo–Fe–Cr thin-film samples composed of multiple uniform compositions were examined by combining corrosion testing with film-thickness evaluation by X-ray fluorescence (XRF). By determining the amount of corrosion from the film thickness before and after corrosion testing, this study aimed to efficiently identify promising composition regions for corrosion resistance in supercritical oxidizing environments.

## 2. Materials and Methods

### 2.1. Combinatorial Deposition

**Figure 1:**
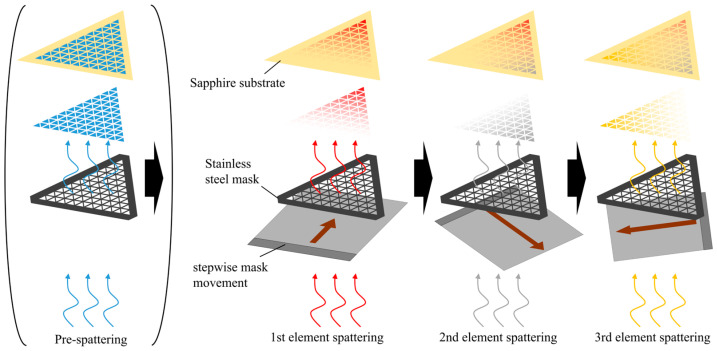
Preparation procedure of a thin-film library with a triangular compositional region by combinatorial deposition.

The combinatorial deposition method enables the fabrication of a large number of compositionally varied thin films in a single run. In this method, a stainless-steel mask with apertures is placed over the substrate, and the deposited amount is controlled by moving the mask (Figure 1). In this study, instead of a continuously graded composition, the substrate was divided into discrete areas, each containing a thin film with a uniform composition. The set of alloy thin films formed on a single substrate is referred to as a “library.” Unlike continuously graded composition-spread films, the present libraries were designed as discrete composition regions formed by stepwise mask movement, in order to suppress strong in-plane composition gradients within each measured region.

Combinatorial deposition was carried out by magnetron sputtering using a combinatorial sputtering system (COMET, CMS-6400, Comet Inc., Tsukuba, Japan). The chamber was evacuated to below 1.0 × 10^−5^ Pa, followed by introduction of Ar to 0.3 Pa. The sputtering gas flow rate was set to 40 sccm (standard cm^3^/min). High-purity elemental targets (99.99%) were used, and deposition was performed at room temperature. For deposition of triangular compositional regions, one element was deposited first, followed by rotation of the substrate by 120° and 240° to deposit the other elements. For the Ru–Ru50Mo50–Ru50W50 library, deposition was sequentially carried out in the order of Ru, W, and Mo. For the Ru60Mo40–Mo40Fe60–Mo40Cr60 library, deposition was carried out sequentially in the order of Mo, Ru, Fe, and Cr. Thin films were deposited on (001) sapphire substrates with a size of 20 × 20 mm. The as-deposited samples were annealed at 400 °C in Ar to promote homogenization. The substrates were then diced into smaller pieces to fit into the supercritical reactor.

### 2.2. Pre-Characterization of Thin-Film Libraries

The deposited libraries were first characterized by X-ray diffraction (XRD) to identify the crystal structures of the thin films (Bruker, Karlsruhe, Germany, D8 Discover). A Cu X-ray source was used with an accelerating voltage and current of 50 kV and 10 mA, respectively. A 1 mm incident slit and a two-dimensional detector (Bruker, Billerica, MA, USA, EIGER2R-500K) were employed. The 2θ range was 20–100°, with a step size of 0.02°.

The chemical composition and film thickness were simultaneously measured by X-ray fluorescence (XRF) (Bruker, Berlin, Germany, M4 TORNADO) at 50 kV and 300 μA over an area of 20 μm in diameter. Film thickness was also evaluated using a stylus profilometer (Surf Corder ET200A, Kosaka Laboratory Ltd., Tokyo, Japan; vertical resolution 0.1 nm), and the relationship between composition and initial thickness obtained by XRF was corrected accordingly. The combined use of XRF and stylus profilometry for thin-film thickness evaluation has been reported previously for sputter-deposited and evaporated metal films [34]. A diamond stylus with a tip radius of 2 μm (ET-1479, Kosaka Laboratory Ltd.) was used. For the Ru–Ru50Mo50–Ru50W50 library, the thickness of all 21 regions was individually measured. In contrast, for the Ru60Mo40–Mo40Fe60–Mo40Cr60 library, thickness was measured only at representative points, and the resulting constant correction factor of 0.8333 was used as a first-order approximation; therefore, the absolute thickness values in this library carry greater uncertainty than those in the Ru–Ru50Mo50–Ru50W50 library.

### 2.3. Combinatorial Supercritical Corrosion Test

**Figure 2:**
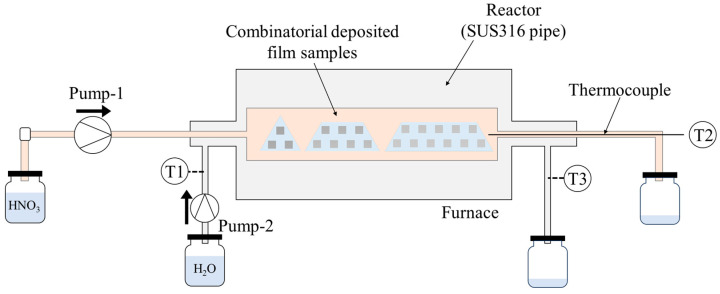
Schematic illustration of the supercritical corrosion test. T1–T3 each denote a thermocouple.

The diced thin-film samples were placed in a SUS316 reactor with an inner diameter of 8.35 mm and connected to a high-pressure pump (Figure 2). First, purified water was flowed through the reactor while heating to 400 °C using a tubular furnace installed around the reactor. After reaching 400 °C, a 0.2 M nitric acid aqueous solution (pH~1) was introduced at a flow rate of 0.5 mL/min, and the pressure was increased to 30 MPa. The system was maintained at 400 °C and 30 MPa for 3 h. Subsequently, the solution was switched back to purified water, and the furnace was turned off to allow cooling.

After the test, the samples were carefully removed from the reactor, rinsed with purified water and isopropyl alcohol, and dried. The corroded samples were then reanalyzed by XRF to determine the composition and film thickness. The corrosion rate R (mm/y) was calculated as

$$R=\frac{t_{prof}^{0}-\alpha t_{XRF}^{post}}{\tau }\times 365\times 24\times 10^{-6}$$

where $t_{prof}^{0}$ is the initial film thickness measured by profilometry (nm), $t_{XRF}^{0}$ is the initial film thickness measured by XRF (nm), $t_{XRF}^{post}$ is the post-corrosion film thickness measured by XRF (nm), τ is the exposure time (h), and α is the thickness correction factor given by $\alpha =t_{prof}^{0}/t_{XRF}^{0}$.

Measurement positions were selected at the centroid of each region whenever possible. Based on prior verification, the film thickness within each region was essentially uniform within the measurement error of the stylus profilometer, except for the peripheral zone (approximately 0.1 mm wide) near the metal-mask edge. In severely damaged regions, post-test thickness was evaluated on areas where the film remained. No independent replicate corrosion tests were performed for identical compositions in the present study, because the primary objective was to establish a first-stage combinatorial screening methodology across a broad composition range.

### 2.4. Evaluation of Corrosion Products

Corrosion products formed after the corrosion test were analyzed by X-ray photoelectron spectroscopy (XPS) (Shimadzu, Kyoto, Japan, KRATOS AXIS-Ultra DLD). An Al Kα X-ray source was used, with an accelerating voltage of 15 kV and an emission current of 10 mA. The analysis area was 110 μm in diameter. The analyzer pass energy was set to 160 eV for survey spectra and 20 eV for high-resolution spectra. The obtained XPS spectra were analyzed using CasaXPS (Casa Software Ltd., Teignmouth, UK, Ver. 2.3.22PR1.0).

## 3. Results

### 3.1. Characterization of Deposited Films

**Figure 3:**
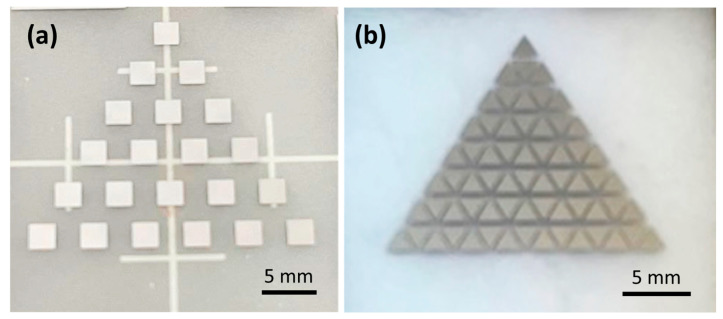
Photographs of thin-film libraries prepared by combinatorial deposition: (**a**) Ru–Ru50Mo50–Ru50W50 and (**b**) Ru60Mo40–Mo40Fe60–Mo40Cr60.

Photographs of the Ru–Ru50Mo50–Ru50W50 and Ru60Mo40–Mo40Fe60–Mo40Cr60 libraries obtained by combinatorial deposition are shown in Figure 3a and b, respectively. In the Ru–Ru50Mo50–Ru50W50 library (Figure 3a), 21 square regions with a size of 2.5 × 2.5 mm were obtained. In the Ru60Mo40–Mo40Fe60–Mo40Cr60 library (Figure 3b), 64 triangular regions with a side length of 1.8 mm were formed. In both cases, the films were dense, well-adhered to the substrate, and exhibited metallic luster.

**Figure 4:**
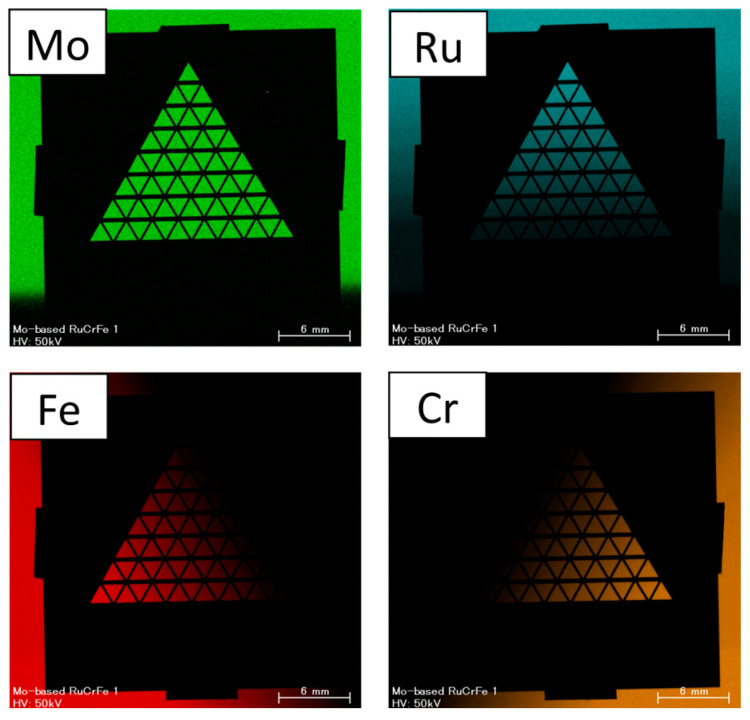
Elemental intensity maps obtained by XRF for the Ru60Mo40–Mo40Fe60–Mo40Cr60 library.

Figure 4 shows the elemental intensity maps obtained by XRF for the Ru60Mo40–Mo40Fe60–Mo40Cr60 library. While Mo was uniformly distributed, the concentrations of Ru, Fe, and Cr gradually decreased from their respective vertices, where each element reached its maximum concentration.

Regarding the compositional analysis by XRF, 0.28 at.% W and 0.81 at.% Mo were detected in the Ru thin film corresponding to the vertex composition. The measured composition for Ru50Mo50 was Ru47.99Mo51.83W0.19, and that for Ru50W50 was Ru52.60Mo0.39W47.02. These deviations may be attributed to measurement uncertainty in XRF as well as slight contamination during deposition. In addition, the Ru sub-peak overlaps with the Mo-Lα peak in energy and is located close to the W-Lα and W-Lβ peaks, which may have affected the quantitative analysis by XRF. Assuming no contamination in the vertex compositions, the maximum compositional error was estimated to be approximately ±3 at.%. If the major component (50 at.%) deviates by ±3 at.%, the uncertainty in film thickness for a nominal thickness of 300 nm was estimated to be approximately ±20 nm. Because the corrosion amount was calculated from thickness differences, the influence of this uncertainty on the corrosion rate maps was reduced, although not eliminated.

**Figure 5:**
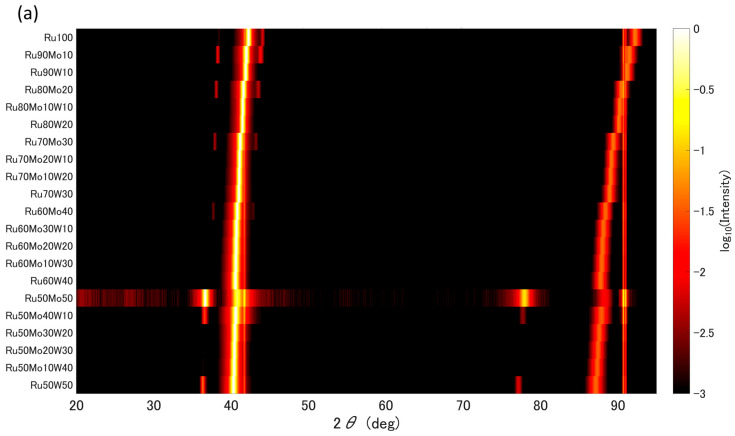

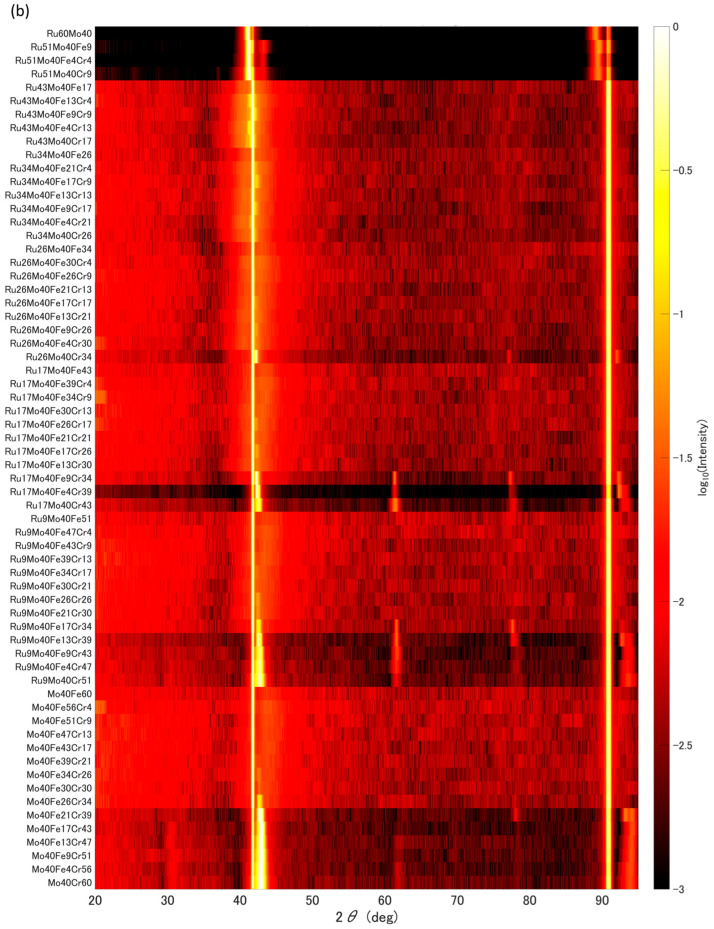
Maps of XRD patterns obtained from the combinatorial libraries. Intensities are shown on a logarithmic scale. (**a**) Ru–Ru50Mo50–Ru50W50; (**b**) Ru60Mo40–Mo40Fe60–Mo40Cr60.

Figure 5a,b show maps of the XRD patterns obtained for each library. The intensity of each pattern was normalized to its maximum value and displayed on a logarithmic scale. Peaks commonly observed at 2θ = 41.7° and 90.7° were attributed to the (006) and (0012) reflections of the Al_2_O_3_ substrate, respectively. In addition, weak side peaks originating from the Cu Kα2 radiation were observed in some patterns.

From the results of the Ru–Ru50Mo50–Ru50W50 library shown in Figure 5a, all films were identified as having a hexagonal close-packed (HCP) structure (space group: P6_3_/mmc). The diffraction peak near 2θ ≈ 38° was assigned to the Ru (100) reflection, that near 2θ ≈ 41° to the Ru (002) reflection, and those in the range of 2θ ≈ 88–92° to the Ru (004) reflection. These peaks shifted toward lower angles with increasing Mo and W content, indicating lattice expansion due to solid-solution formation. Additional diffraction peaks corresponding to the Ru (101) and (103) reflections were also observed; however, the films were found to be preferentially oriented along the (001) direction.

In the Ru60Mo40–Mo40Fe60–Mo40Cr60 compositional system, the (002) reflection attributable to the HCP Ru solid solution was observed for compositions containing > 50 at.% Ru, whereas distinct peaks were not often observed at Ru concentrations below 50 at.%, particularly for Fe-rich compositions, indicating amorphization (Figure 5b). For Cr-rich compositions (approximately > 34 at.%), several peaks attributable to the BCC phase (space group: Im-3m) were observed. The peaks at 2θ = 43° and 94° were assigned to the (110) and (220) reflections, respectively, while the weaker peaks at 2θ = 62° and 78° were assigned to the (200) and (211) reflections, respectively [35].

According to the Mo–Fe–Cr ternary phase diagram, at 600 °C, Mo exhibits complete solid solubility with Cr to form a BCC solid solution and can additionally dissolve up to ~22 at.% Fe [36]. As the Cr content decreases, the solubility of Fe in the BCC solid solution decreases to only a few at.%, and the BCC phase coexists in equilibrium with the Laves phase (Fe_2_Mo, space group: P6_3_/mmc) [37]. Therefore, for Fe-rich compositions, sufficient diffusion for the formation of the Laves phase may not have occurred during the relatively low-temperature post-deposition annealing process, resulting in an amorphous or fine crystalline microstructure.

On the other hand, the low background level observed for the Ru solid solution is consistent with the wide solid-solubility range of Ru for the constituent elements. Ru can dissolve up to approximately 50 at.% Mo, 77 at.% Fe, and 51 at.% Cr [38,39,40], suggesting that good crystallinity was maintained.

**Figure 6:**
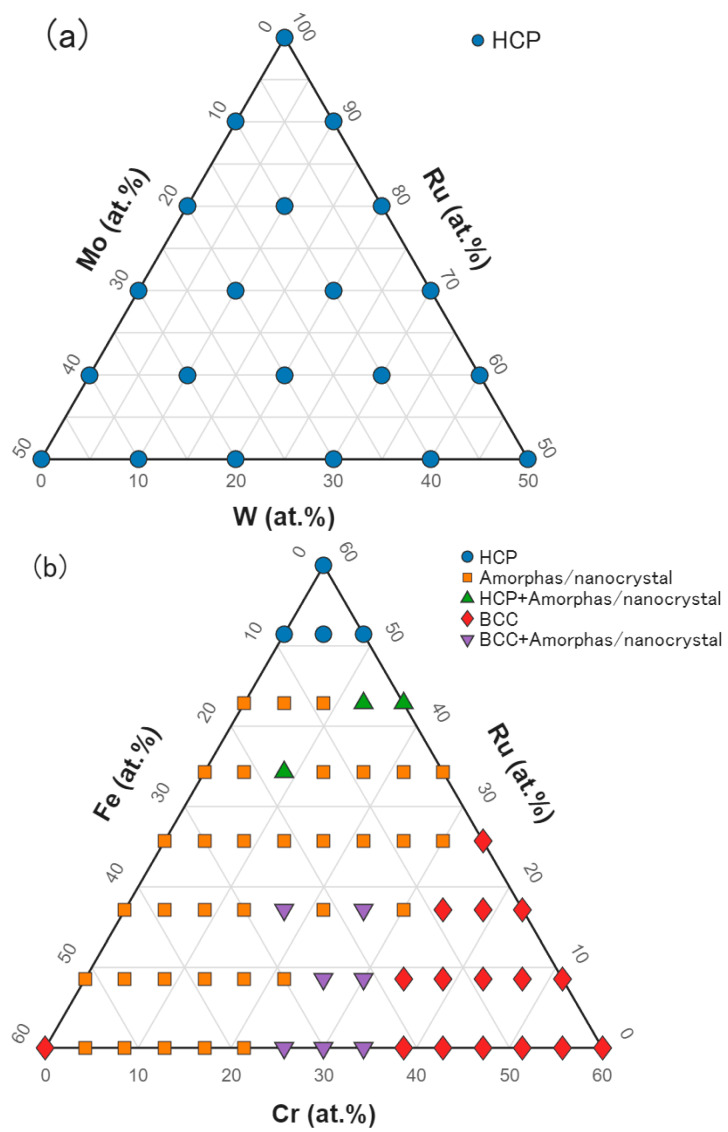
Phase summary maps of the combinatorial libraries: (**a**) Ru–Ru50Mo50–Ru50W50 and (**b**) Ru60Mo40–Mo40Fe60–Mo40Cr60.

Based on the dominant diffraction features, phase summary maps were constructed for the two libraries (Figure 6). The Ru–Ru50Mo50–Ru50W50 library was classified as an HCP Ru-based solid-solution region over the measured composition range, whereas the Ru60Mo40–Mo40Fe60–Mo40Cr60 library contained HCP, BCC, and amorphous/weakly crystalline regions depending on composition.

### 3.2. Corrosion Characteristics

**Figure 7:**
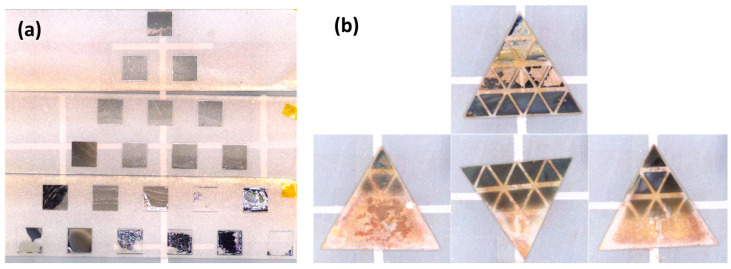
Photographs of thin-film libraries after corrosion testing in supercritical nitric acid: (**a**) Ru–Ru50Mo50–Ru50W50 and (**b**) Ru60Mo40–Mo40Fe60–Mo40Cr60.

Figure 7a,b show photographs of the thin-film libraries after the corrosion test. In the Ru–Ru50Mo50–Ru50W50 system, most regions remained intact and could be evaluated. In contrast, in the Ru60Mo40–Mo40Fe60–Mo40Cr60 system, significant film delamination or near-complete dissolution was observed in compositions on the low-Ru side; however, the film thickness could still be evaluated from the remaining areas.

**Figure 8:**
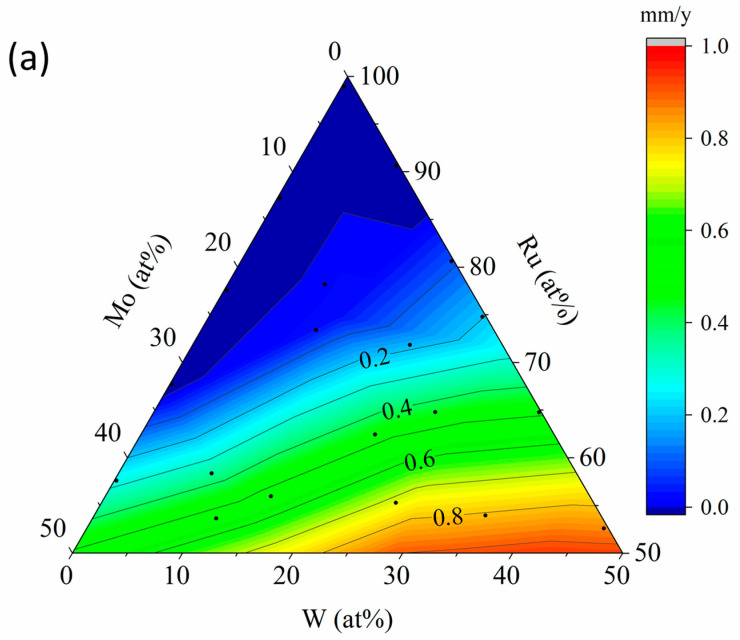

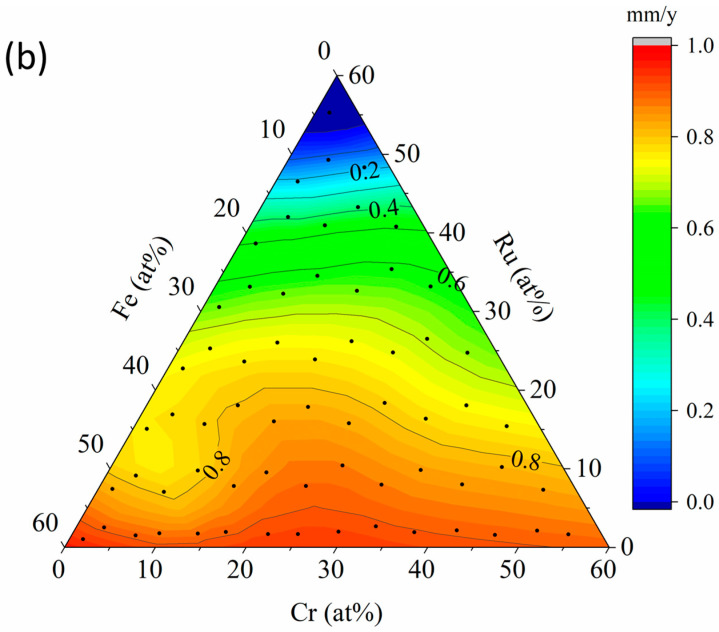
Corrosion rate maps obtained in supercritical nitric acid at 400 °C and 30 MPa. (**a**) Ru–Ru50Mo50–Ru50W50 and (**b**) Ru60Mo40–Mo40Fe60–Mo40Cr60. The black dots in the ternary plots correspond to the measured compositions after corrosion determined by XRF. The low-Ru region includes compositions where severe delamination or near-complete dissolution occurred; the values in this region should be interpreted as qualitative screening indicators.

Figure 8 shows the corrosion rate maps obtained from corrosion tests in supercritical nitric acid. Figure 8a presents the map for the Ru–Ru50Mo50–Ru50W50 system. As indicated by the contour lines, the annual corrosion rate was below 0.2 mm/y over most of the compositional range. The contour distribution changed monotonically mainly along the Ru concentration. In the Mo-added system, good corrosion resistance was obtained at Ru concentrations of approximately 60 at.% or higher, whereas in the W-added system, this condition was limited to the more Ru-rich region, namely approximately 80 at.% or higher.

A similar trend was also observed in the Ru60Mo40–Mo40Fe60–Mo40Cr60 system, where good corrosion resistance of <0.2 mm/y was obtained for compositions containing approximately >50 at.% Ru (Figure 8b). No clear difference between Fe and Cr was identified within the resolution of the present screening. On the low-Ru side, poor corrosion resistance was observed both in the BCC single-phase region and in the amorphous or weakly crystalline region identified by XRD (cf. Figure 6b).

In the low-Ru region, severe delamination or near-complete dissolution occurred; the implications for the quantitative reliability of the thickness-based evaluation are discussed in Section 4.2.

### 3.3. Surface Characterization by XPS

**Figure 9:**
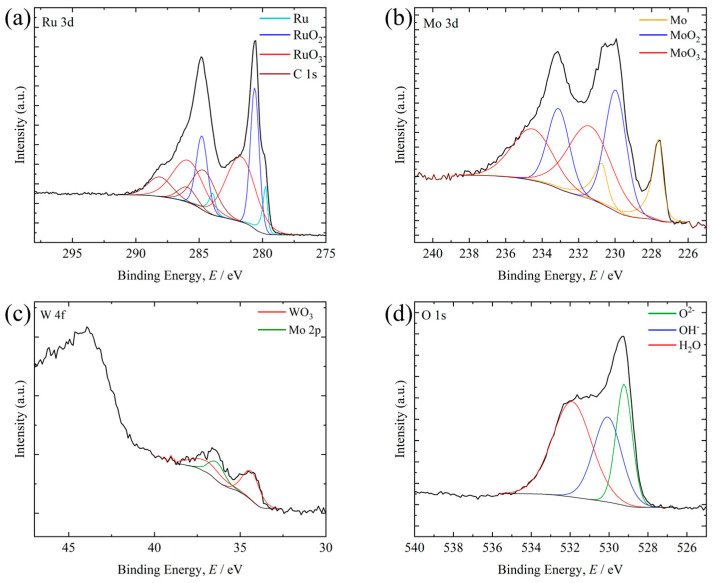
XPS spectra obtained from the surface of the Ru_57.6_Mo_42.2_W_0.2_ after the supercritical HNO_3_ flow test: (**a**) Ru 3d, (**b**) Mo 3d, (**c**) W 4f, and (**d**) O 1s.

**Figure 10:**
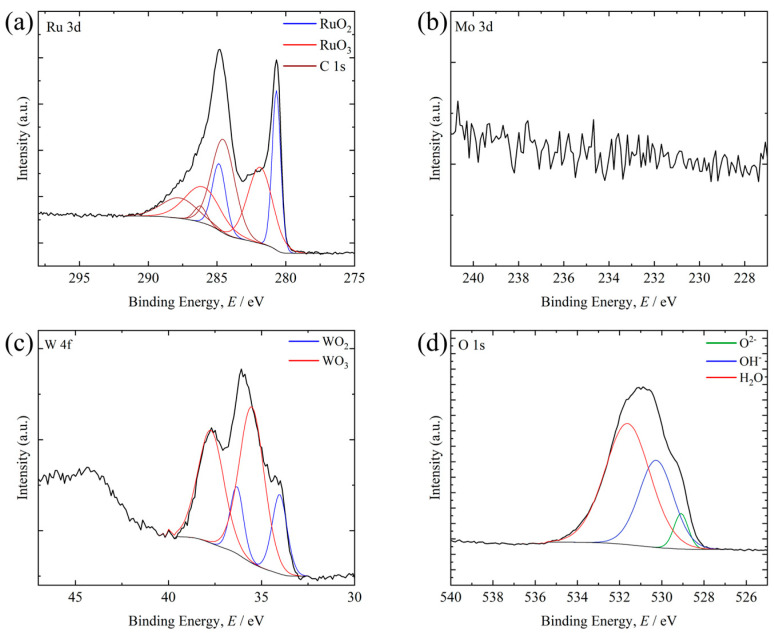
XPS spectra obtained from the surface of the Ru_64.8_Mo_0.2_W_35.0_ after the supercritical HNO_3_ flow test: (**a**) Ru 3d, (**b**) Mo 3d, (**c**) W 4f, and (**d**) O 1s.

**Figure 11:**
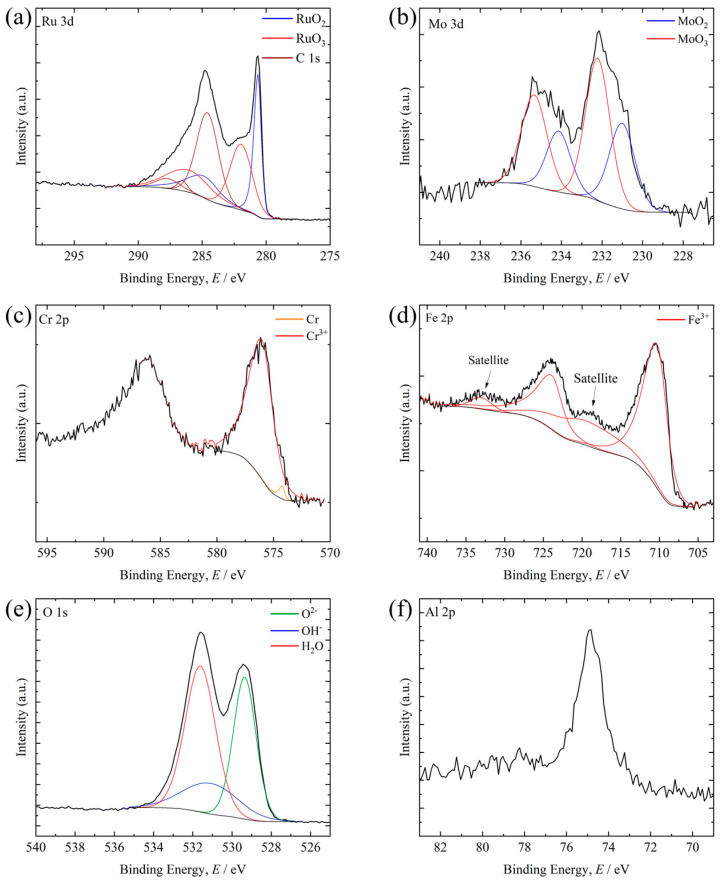
XPS spectra obtained from the surface of the Ru_24.2_Mo_39.2_Cr_18.8_Fe_20.8_ after the supercritical HNO_3_ flow test: (**a**) Ru 3d, (**b**) Mo 3d, (**c**) Cr 2p, (**d**) Fe 2p, (**e**) O 1s, and (**f**) Al 2p.

Figure 9, Figure 10 and Figure 11 show XPS spectra for representative compositions in each library after the supercritical nitric acid flow test. In each figure, the black solid line represents the Shirley-type background. The compositions are the measured values determined by XRF after the corrosion tests. The XPS results were interpreted as reflecting the chemistry of the surviving post-corrosion surface within the analysis depth, rather than the average composition of the entire alloy region.

On the surface of Ru_57.6_Mo_42.2_W_0.2_ (Ru–Mo system), peaks assigned to RuO_2_, RuO_3_, MoO_2_, and MoO_3_ were observed, indicating the formation of an oxide layer containing these species (Figure 9). Peaks assigned to metallic Ru and Mo were also detected. Because the analysis depth of XPS using an Al Kα source is on the order of several nanometers, this result indicates that the oxide layer on Ru_57.6_Mo_42.2_W_0.2_ was relatively thin.

In contrast, on Ru_64.8_Mo_0.2_W_35.0_ (Ru–W system), peaks assigned to RuO_2_, RuO_3_, WO_2_, and WO_3_ were observed, indicating the formation of an oxide layer containing these species (Figure 10). No peaks corresponding to metallic Ru or W were detected, suggesting that a thicker oxide layer formed compared with the Ru–Mo alloy.

Figure 11 shows the XPS spectra of Ru_24.2_Mo_39.2_Cr_18.8_Fe_20.8_. Peaks assigned to RuO_2_, RuO_3_, MoO_2_, MoO_3_, as well as Cr^3+^ and Fe^3+^, were observed, indicating the formation of an oxide layer containing these species. No peaks assigned to metallic Ru or Mo were detected, suggesting the formation of a relatively thick oxide layer. Furthermore, in the Ru–Mo–Cr–Fe system, the peak intensity of MoO_3_ relative to MoO_2_ was higher than in the Ru–Mo system.

## 4. Discussion

### 4.1. Interpretation of the Corrosion Maps

The corrosion maps show that corrosion resistance in both libraries changed mainly with the Ru content. In the Ru–Ru50Mo50–Ru50W50 library, most compositions on the Ru-rich side showed corrosion rates below 0.2 mm/y, whereas corrosion increased as the Ru content decreased. A similar tendency was also observed in the Ru60Mo40–Mo40Fe60–Mo40Cr60 library. These results indicate that a sufficiently high Ru content is the main requirement for corrosion resistance under the present supercritical nitric acid conditions. Conversely, because the initial film thickness was approximately 300 nm, complete film loss during the 3 h exposure would correspond to about 0.88 mm/y; once the thickness loss approaches this level, local thickness-based evaluation loses quantitative meaning, and the affected regions should be interpreted only as indicators of severe corrosion.

At similar Ru contents, the Mo-containing side showed better corrosion resistance than the W-containing side. This tendency is broadly consistent with the XPS results for the representative compositions. Metallic Ru and Mo were still detected on Ru57.6Mo42.2W0.2, whereas only oxide peaks were detected on Ru64.8Mo0.2W35.0, suggesting that the oxide layer was thicker in the W-containing film. Murugesan et al. [26] reported that WO_3_ formed in Ru–Mo–W alloys in sulfuric acid does not necessarily improve corrosion resistance. Although the environment is different, the present corrosion maps and XPS results are consistent with the idea that the WO_3_-containing surface layer was less protective than the surface layer formed on the Ru–Mo composition under the present conditions.

### 4.2. Applicability and Limitations of the Present Method

The present method is intended for the first step of screening corrosion-resistant compositions in supercritical acidic environments. Its main strength is that many compositions can be compared in a limited number of corrosion tests. For this reason, the present maps are useful for identifying clearly promising regions and clearly poor regions, and for selecting compositions for follow-up experiments. Under the present conditions, a corrosion rate of 0.2 mm/y corresponds to a thickness loss of about 68 nm over 3 h, which is larger than the estimated thickness uncertainty of about 20 nm. Therefore, the present condition is suitable for judging whether an intact region is roughly above or below the screening benchmark, although this advantage becomes limited once severe delamination or near-complete dissolution occurs.

Several limitations should also be noted. First, repeated corrosion tests were not carried out for the same composition. Quantitative consistency was checked using Ru-rich, Mo-containing spots close to Ru60Mo40 that were present in both libraries. In the Ru–Ru50Mo50–Ru50W50 library, the closest spot (Ru57.6Mo42.2W0.2) gave R = −0.007 mm/y, while in the Ru60Mo40–Mo40Fe60–Mo40Cr60 library, the closest spot (Ru56.8Mo38.3 with minor Fe and Cr) gave R = −0.003 mm/y. Both values are within the α-derived uncertainty floor (≈±0.05 mm/y) and are mutually consistent, although they are not equivalent to true replicate measurements on the same composition. Second, the two libraries were not treated in the same way when the initial thickness was corrected. In addition, XRF peak overlap and residual compositional uncertainty, especially near the composition extremes, may also affect the absolute values in the corrosion rate maps. In the Ru–Ru50Mo50–Ru50W50 library, all regions were measured by profilometry, whereas in the Ru60Mo40–Mo40Fe60–Mo40Cr60 library, a correction factor based on representative points was used. Therefore, the latter library is better suited to showing overall trends than to the precise comparison of neighboring compositions.

Third, in regions where severe delamination or near-complete dissolution occurred, post-test thickness had to be measured on areas where the film remained. In such regions, the corrosion rates should be treated as approximate values. This is why the low-Ru region is interpreted mainly as a region of poor corrosion resistance, rather than as a detailed ranking of compositions. In addition, species dissolved from the SUS316 reactor may have affected the post-test surface condition. At the Ru60Mo40 vertex composition, post-corrosion XRF measurements did not show a clear increase in Fe or Cr beyond the uncertainty range of the XRF analysis, and thus no clear evidence of substantial redeposition from the reactor was obtained for this composition. For more reliable measurements, future work should use a liner or reactor material that does not contain the elements being evaluated in the present alloy systems.

Finally, the thin-film libraries used here are not equivalent to bulk alloys. This point is consistent with recent work showing that thin-film libraries are useful for identifying relative compositional trends, whereas the quantitative passivation behavior can differ from that of bulk alloys [33]. Through-thickness compositional uniformity was not directly examined, and the actual surface composition during corrosion may differ from the XRF-measured composition because of surface enrichment or depletion. In addition, XPS only probes the near-surface region of the remaining film. For this reason, thicker films or bulk specimens, together with depth-profile measurements, will be needed in future work.

Cross-sectional microscopy, quantitative adhesion measurements, ICP analysis of single-composition follow-up tests, and XPS depth profiling will be useful in future work to clarify interface degradation and oxide-scale structure.

## 5. Conclusions

In this study, Ru-based alloy thin-film libraries were fabricated using a combinatorial deposition method, and their corrosion resistance was evaluated in a flowing supercritical nitric acid environment at 400 °C and 30 MPa. The main conclusions are summarized as follows:

(i) A first-stage high-throughput screening method for corrosion resistance under supercritical conditions was established by combining XRF-based simultaneous evaluation of composition and film thickness with correction using profilometer-derived initial thickness.
(ii) In the Ru–Ru50Mo50–Ru50W50 library, most compositions exhibited corrosion rates below 0.2 mm/y. At comparable Ru contents, higher Mo concentrations provided better corrosion resistance than W.
(iii) In the Ru60Mo40–Mo40Fe60–Mo40Cr60 library, significant dissolution and film delamination were observed in compositions with Ru contents below approximately 50 at.%.
(iv) X-ray photoelectron spectroscopy (XPS) analysis of post-corrosion surfaces revealed that Ru oxides were predominantly formed in representative corrosion-resistant compositions, while in the Ru–Mo–Fe–Cr system, Mo oxides were also detected.

These results identify promising composition ranges of Ru-based alloys for supercritical oxidizing acidic environments and show that the present combinatorial deposition approach is a useful first-stage tool for screening candidate compositions under such severe conditions. To improve quantitative reliability and extend the range of compositions that can be examined, improvements in film adhesion, film crystallinity, and reactor corrosion resistance will be beneficial. Further validation using thicker films or bulk specimens will be necessary to establish design guidelines for practical applications.

## Data Availability

The original contributions presented in this study are included in the article. Further inquiries can be directed to the corresponding author.
